# Feasibility of collecting and assessing patient-reported outcomes for emergency admissions: laparotomy for gastrointestinal conditions

**DOI:** 10.1136/bmjgast-2018-000238

**Published:** 2018-10-16

**Authors:** Esther Kwong, Jenny Neuburger, Dave Murray, Nick Black

**Affiliations:** 1 Departement of Health Services Research and Policy, London School of Hygiene and Tropical Medicine, London, UK; 2 Anaesthetic Department, James Cook University Hospital, Middlesbrough, UK

**Keywords:** patient reported outcome measures, health status, health-related quality of life, retrospective, response rate feasibility, emergency admissions, emergency laparotomy

## Abstract

**Introduction:**

Audit of emergency surgery is usually limited to immediate clinical outcomes relating to outcomes during the acute hospital episode with little attempt to capture patients’ views of their longer-term outcomes. Our aim was to determine the response rate to patient-reported outcome measures (PROMs) for patients who underwent an emergency laparotomy for gastrointestinal conditions, identify response bias and explore the feasibility of comparing outcomes with their prior health based on their recalled view collected during their admission.

**Methods:**

Patients undergoing emergency laparotomy in 11 hospitals were recruited to complete a retrospective questionnaire containing the EQ-5D-3L and Gastrointestinal Quality of Life Index (GIQLI). Response rate for 3-month mailed follow-up questionnaire and potential response biases were assessed. Patients’ outcomes were compared with their baseline using χ^2^ and paired t-test to assess for differences.

**Results:**

Of 255 patients contacted at 3 months, 190 (74.1%) responded. Responders were more likely to be older, female and more affluent. Patients’ health improved significantly as regards the GIQLI (93.3 vs 97.9; p=0.048) and the subscale on symptoms (51.9 vs 59.6; p<0.001). No significant change in subscales on emotion or physical aspects or for overall health status (EQ-5D: 0.58 vs 0.64; p=0.06). According to the social subscale, patients had deteriorated (11.0 vs 9.8; p<0.0006). Differences in change scores by patient characteristics were slight, suggesting minimal response bias.

**Conclusion:**

This approach offers the opportunity for assessing the impact of treatment, from the patient’s perspective and the potential to evaluate emergency laparotomy care using PROMs.

**Summary box**

**What is already known about this subject?**

- Laparotomy is one of the most common emergency surgical interventions with higher postoperative morbidity and mortality than elective procedures.
- In elective surgery, these outcomes can be supplemented by patient-reported outcome measures (PROMs), but they have not been used routinely for emergency admissions.
- While the feasibility of asking emergency laparotomy patients to recall their pre-admission health status has been demonstrated, their likelihood of responding to a mailed postdischarge questionnaire is unknown.

**What are the new findings?**

- PROMs can be successfully collected in patients 3 months after emergency laparotomy with a response rate of 74% using mailed follow-up.
- Most patients have regained their prior level of gastrointestinal health and their general health also improved.

**How might it impact on clinical practice in the foreseeable future?**

- PROMs offer the opportunity for routinely assessing the impact of treatment from the patient’s perspective.
- Meaningful comparisons of surgeons and hospitals based on PROMs could be undertaken to supplement clinical measures such as mortality, morbidity and complications.

## Introduction

In England, 40% of National Health Service (NHS) hospital admissions and 18% of procedures are emergencies.^1 2^ In 2016/2017, there were about 1.96 million procedures conducted by general surgeons for digestive tract conditions (excluding appendicectomy) of which 116 000 (6%) were emergency operations.^3^ Emergency laparotomy has a higher postoperative morbidity and mortality than elective procedures.^4^

If the aim of healthcare is to restore a patient to his or her full potential, we need to be able to compare patients’ outcomes with their health status before the sudden and unexpected event that led to their emergency admission. Patient-reported outcomes measures (PROMs) are one of the ways to measure effectiveness and to determine the benefit of resources spent.^5 6^ PROMs are self-reported questionnaires designed to be completed by patients to capture their health at specific points in time to detect a health change over a period. They are multidimensional measures which may cover symptoms, functional status or health-related quality of life.^6^

It is known that short-term clinical outcomes, such as morbidity and mortality, following emergency surgical care vary significantly between hospitals.^7 8^ In contrast, little is known about the longer-term health status of those who survive who make up the vast majority of patients. Capturing PROMs would provide an additional means of routinely assessing the effectiveness of emergency surgical care. Currently, we know little about whether PROMs for emergency surgery vary between hospitals and whether there is any unwarranted variation.

There is minimal existing research about the feasibility of collecting routine follow-up PROMs from patients who have completed a PROM during their inpatient episode. The relevance of the available evidence is unclear as studies either involved only a few centres or were restricted to protocol-driven intervention trials instead of routine use.^9 10^ In addition, studies were mostly conducted in other countries, so the results may not be applicable in England.^11–18^ Response rates ranged between 51% and 71% for mailed questionnaires, and between 51% and 84% for interviewer-administered questionnaires. The only attempt in England to collect PROMs in multiple sites involved 28 major trauma centres and achieved about a 50% response rate using mailed or online follow-up at 6 months (personal communication: Antoinette Edwards).

The reliability of patients’ recalling their prior health status via the use of a retrospective PROM has been demonstrated in six studies mostly in the USA.^19 20^ To determine the feasibility of employing PROMs in emergency admissions, we undertook two exploratory studies, one on a medical condition and the other in surgery (emergency laparotomy). Patients’ recollected state of health prior to their admission was collected shortly after their laparotomy but before discharge from hospital to provide a baseline assessment. The feasibility of recruiting representative samples of patients has already been reported.^21^

This paper reports on the follow-up response rate for patients, identifies any response biases and explores the feasibility of comparing patients’ outcome at 3 months with their retrospectively collected PROMs at baseline.

## Methods

### Site and patient recruitment

A multisite study was carried out to ensure there would be variation in the administration of patient recruitment and data collection. This would allow us to gain insights into the relative merits of recruiting in different settings and with different personnel involved. Fourteen hospitals were selected, on the basis of their high case ascertainment rates in the National Emergency Laparotomy Audit (NELA), of which 13 agreed to participate and 11 successfully recruited patients for the 15-week duration of the study.

Patients who met the NELA inclusion criteria and were alive at discharge were eligible for inclusion in this study unless they were not literate in English, deemed not to have sufficient cognitive ability or were not residents in the UK. For NELA, all patients over the age of 18 years, having a general surgical emergency laparotomy in all NHS hospitals in England and Wales, are eligible for inclusion and are enrolled on a prospective basis into the audit. The inclusion criteria for the audit aims to include all emergency gastrointestinal procedures on the stomach, large and small bowel, for conditions such as perforation, bleeding, abdominal abscess or obstruction, via open or laparoscopic approaches. Emergency laparotomies following elective surgical complications are also included. Patients requiring vascular surgery, gynaecological surgery, surgery on the renal tract, appendicectomy for appendicitis and laparotomy following trauma are excluded from the audit.^22^

Patients were invited to participate after surgery, before discharge and as close to the discharge date as possible to ensure the immediate effects of the intervention (such as a general anaesthetic and immediate postoperative complications including ileus, respiratory depression and side effects of opioids) were minimised to ensure that the patients were medically able to complete the questionnaire.^21^ Clinical staff explained the study to patients, provided written information and obtained written consent. Questionnaires recalling their pre-admission baseline health status were completed by patients without assistance from staff or family except when they were impeded by physical disability or sensory impairment.

### Three-month follow-up

Patients were mailed a follow-up questionnaire (QF) from the London School of Hygiene and Tropical Medicine 12 weeks (84 days) after their date of admission to hospital. Patient vital status was first checked against the Personal Demographics Service at NHS Digital prior to sending a follow-up questionnaire. After 2 weeks, non-responders were sent a reminder questionnaire.

### Questionnaires

The questionnaires completed during the admission included demographic information, self-reported comorbidities, a disease-specific PROM and a generic PROM. Patients were asked to recall how they were a month before their current admission. A systematic review identified suitable PROMs with adequate psychometric properties. Clinicians were then consulted in an unstructured meeting (a formal consensus development method was not used) to determine the final choice. This included consideration of the length and likely burden on patients of instruments.

The disease-specific PROM was the Gastrointestinal Quality of Life Index (GIQLI), developed by Eypasch and colleagues.^23^ It consists of 36 questions relating to the gastrointestinal system and the impact of symptoms and treatment on individuals’ physical, emotional and social status. It takes 5–10 min to complete and has good test–retest reliability (intraclass correlation coefficient 0.92) and internal consistency (Cronbach’s alpha >0.90). The GIQLI is the most commonly used validated GI system–specific PROM for studies investigating outcomes in emergency abdominal surgery. The GIQLI score provides a global index score from 0 (poor health) to 144 (excellent health). The index score comprises four subscales: GIQLI symptoms (0–76), GIQLI physical score (0–28), social score (0–16) and emotion score (0–20) (online supplementary appendix 1). One item, on sex life, may not be applicable for some patients, but the option of such a response is not available. Despite this, some patients wrote ‘not applicable’ on their questionnaire. They were coded as ‘not at all’.

10.1136/bmjgast-2018-000238.supp1 Supplementary data

A generic PROM was also included as it allows for comparisons across conditions and treatment groups and the potential to derive quality-adjusted life years for economic evaluations. The generic PROM used was the EQ-5D-3L, which has five items: mobility, usual activities, personal care, pain/discomfort and anxiety/depression. It takes up to 5 min to complete.^24^ For each of these questions, the respondent chooses from three responses indicating the level of their function. A multi-attribute utility score where death and perfect health are represented by 0 and 1 are calculated.^25^ Scores less than 0 are considered worse than death and one is the maximum score possible. The EQ-5D-3L was used rather than the EQ-5D-5L as the former is still the version used in the National PROMs Programme in England.

### Analysis

Participating patients’ characteristics were summarised using means and SDs for continuous variables or percentages for binary variables. Response rates were calculated and reported for patients grouped by age, sex, living arrangements, socioeconomic status (SES), baseline GIQLI scores and baseline EQ-5D scores. SES was measured using the English Index of Multiple Deprivation (IMD) based on patients’ residential postcodes^26^ with patients assigned to quintiles of the national ranking of IMD scores.

We conducted χ^2^ and paired t-test for differences to compare characteristics of participants who responded to the 3-month QF with those who did not. Patients’ outcomes at 3 months were compared with their baseline using paired t-test to assess evidence of change in health status. Change scores, with the 95% CI, were also used to describe reasonable limits on the extent of any change in order to assess whether the results were consistent with recovery to baseline (no change or an improvement in scores).

Using the characteristics of those who were more likely to respond, we compared baseline and follow-up scores in these groups and quantified the potential impact this would have had on the mean health change. We then calculated what the health change would have been should the response rate be the same as recruitment proportions for the patient characteristics shown have a statistically significant non-response association. These calculations are estimations based on the assumption that non-responders would have reported similar PROM changes as responders, to help quantify the extent of influence non-response has on the change in PROM scores as an illustration of the potential impact.

## Results

### Response rates

A total of 268 patients were recruited and completed baseline questionnaires (online supplementary appendix 2). Of these, 13 (4.9%) patients who were discharged from hospital then died during the postdischarge period before the follow-up contact. Of the 255 survivors, 190 patients (74.1%) responded to the follow-up PROM questionnaire: 146 responded to the first request and 44 after one reminder.

The mean time between completing the baseline (Q1) and the QF was 85 (SD 19) days, and between admission and QF, 94 days.

### Response bias

**Figure 1:**
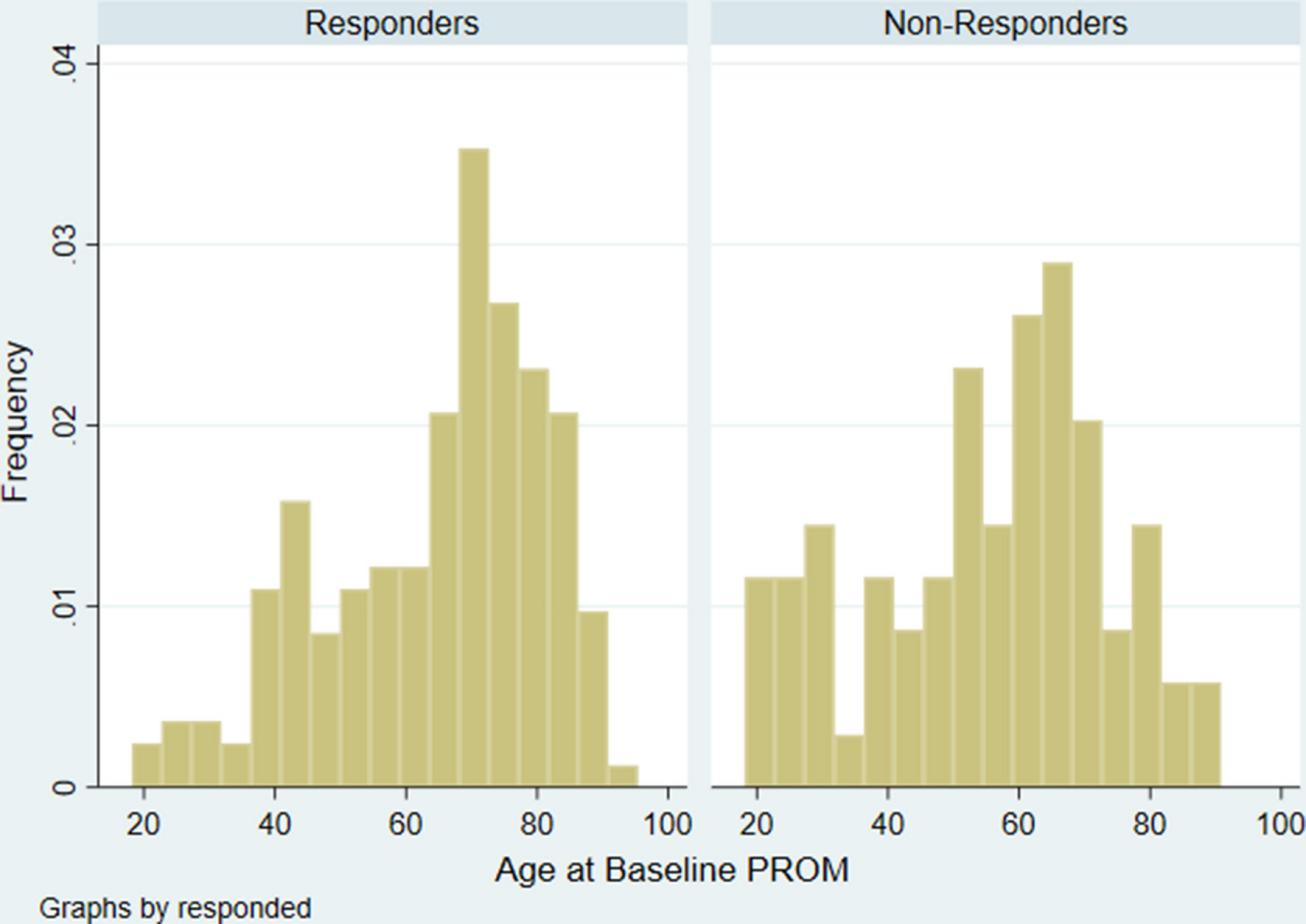
Age distribution of responders and non-responders. PROM, patient-reported outcome measure.

Responders and non-responders were similar as regards comorbidities, living arrangements and health status (EQ-5D and GIQLI) (table 1). Responders differed from non-responders in three ways: they were older (mean age 65.0 (SD 16; range 18–91) vs 53.4 (SD 18; range 19–88); p<0.0001) (figure 1), more likely to be women and more likely to come from more affluent SES.

**Table 1:** Characteristics of responders compared with non-responders

Patient characteristic	Overall (n=255)	Responders (n=189)	Non-responders (n=66)	P values*
Sex				
Male	118 (46.0)	80 (42.3)	38 (57.6)	0.03
Female	137 (54.0)	109 (57.7)	28 (42.4)	
SES				
1 (least deprived)	34 (14.8)	29 (17.1)	5 (8.3)	0.03
2	47 (20.4)	37 (21.0)	10 (16.7)	
3	49 (23.3)	38 (22.3)	11 (18.3)	
4	49 (21.3)	37 (21.8)	12 (20.0)	
5 (most deprived)	51 (22.2)	29 (17.6)	22 (36.7)	
Missing	25	19	6	
Comorbidities				
0	58 (24.2)	37 (21.2)	21 (33.3)	0.186
1	78 (32.6)	64 (36.4)	14 (22.2)	
2	44 (18.4)	33 (18.7)	11 (17.5)	
3	32 (13.4)	22 (12.5)	10 (15.9)	
4 or more	27 (11.3)	20 (11.4)	7 (11.1)	
Missing	16	13	3	
Living arrangements				
With family	203 (79.6)	149 (82.3)	54 (84.3)	0.685
Alone	40 (15.7)	30 (16.5)	10 (15.6)	
Other	2 (0.8)	2 (1.1)	0	
Missing	10	8	2	
Mean EQ-5D (SD)	0.57 (0.40)	0.58 (0.39)	0.54 (0.42)	0.494
Missing	12	10	2	
Mean GIQLI (SD), g	94.1 (31.3)	94.7 (31.4)	92.3 (31.04)	0.619
Missing	25	18	7	

*From χ^2^.

GIQLI, Gastrointestinal Quality of Life Index; SES, socioeconomic status.

### Comparing change in PROM scores

**Figure 2:**
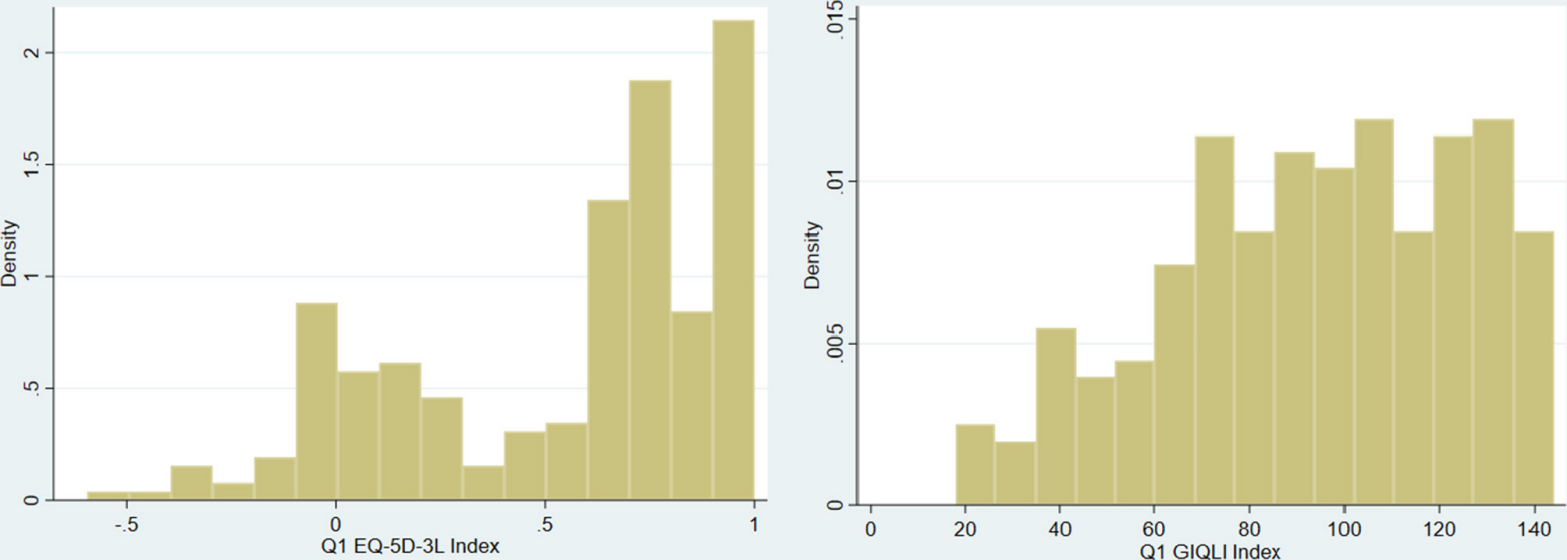
Baseline Gastrointestinal Quality of Life Index (GIQLI) score and baseline EQ-5D score distributions.

The distribution of the EQ-5D at baseline was bimodal, with the majority of patients above 0.5 and a smaller peak between −0.5 and 0.5 (figure 2). The distribution of the GIQLI score was broadly normal with a left skew.

Three months after surgery, patients’ GIQLI emotion score and GIQLI physical score had returned to the baseline score (table 2). The GIQLI symptom score had improved (51.9 vs 59.6; p<0.001), whereas the GIQLI social score had deteriorated (11.2 vs 9.8; p<0.001).

**Table 2:** Comparison of baseline and follow-up PROM scores

PROM	Number with complete data	Baseline (Q1) Mean (SE, 95% CI)	Follow-up (QF) Mean (SE, 95% CI)	Change (95% CI, p values)
GIQLI	158	93.3 (2.55, 88.3 to 98.4)	97.9 (1.77, 94.4 to 101.4)	+4.6 (0.37 to 8.83, 0.048)
GIQLI symptom	168	52.0 (1.18, 49.6 to 54.2)	59.5 (0.76, 58.0 to 61.0)	+7.5 (5.68 to 9.32, <0.0001)
GIQLI emotion	177	12.0 (0.45, 11.12 to 12.9)	12.3 (0.35, 11.6 to 13.0)	+0.3 (–0.43 to 1.04, 0.37)
GIQLI physical	176	14.0 (0.61, 12.8 to 15.2)	13.3 (0.46, 12.4 to 14.2)	−0.7 (–1.68 to 0.28, 0.18)
GIQLI social	174	11.0 (0.34, 10.4 to 11.7)	9.8 (0.29, 9.27 to 10.4)	−1.2 (–1.82 to –0.58, 0.0006)
EQ-5D index	175	0.58 (0.03, 0.52 to 0.64)	0.64 (0.03, 0.59 to 0.69)	+0.06 (0.00 to 0.12, 0.06)

GIQLI, Gastrointestinal Quality of Life Index; PROM, patient-reported outcome measure; QF, follow-up questionnaire.

The GIQLI score had improved (93.3 vs 97.9, p=0.048) and EQ-5D score had improved considerably (0.58 vs 0.64), although this difference was not quite statistically significant (p=0.06).

### Influence of non-response on change in health status

Change in the GIQLI score and in the EQ-5D score was not associated with patients’ SES (table 3). However, change was greater in younger (under 70 years) and female patients though the differences did not reach statistical significance except for EQ-5D in women.

**Table 3:** Change in PROMs scores by age, sex and SES

Patient characteristic	Change in GIQLI (SD) (n=158)	P values*	Change in EQ-5D (SD) (n=160)	P values*
Age (years)				
>70	1.5 (25.0)	0.46	0.03 (0.36)	0.70
50–70	6.9 (25.8)		0.07 (0.37)	
<50	7.8 (39.3)		0.09 (0.50)	
Sex				
Male	2.46 (28.7)	0.43	−0.01 (0.40)	0.047
Female	6.14 (29.3)		0.11 (0.39)	
SES				
1 (least deprived)	2.39 (23.7)	0.69	0.13 (0.32)	0.61
2	–0.75 (24.4)		0.47 (0.32)	
3	3.94 (28.1)		0.04 (0.39)	
4	4.52 (26.4)		0.11 (0.42)	
5 (most deprived)	9.96 (27.5)		–0.01 (0.49)	

*From ANOVA.

GIQLI, Gastrointestinal Quality of Life Index; PROM, patient-reported outcome measure; SES, socioeconomic status.

### Assessment of non-response bias

Assessment of potential biases that might have been introduced by some patients not responding was based on the assumption that patients with similar baseline characteristics (sex and age) would have had similar follow-up EQ-5D or GIQLI scores. To illustrate the impact on non-response linked to sex and age, we estimated the mean change in GIQLI and EQ-5D scores had there been 100% follow-up response rate, compared with the observed mean changes. With this assumption, if responses were as per recruitment proportions by gender, the change in GIQLI would have been 4.55 (for all participants including non-responders) compared with 4.60 (observed in responders) and the mean change in EQ-5D would have been 0.055 compared with the observed mean change of 0.060.

If responses were as per recruitment proportions by age, the change in GIQLI would have been 5.10 instead of 4.60, and the mean change in EQ-5D would have been 0.061 instead of 0.060.

## Discussion

### Main findings

Retrospective and 3-month follow-up PROMs can be successfully collected in representative samples of patients undergoing emergency laparotomy surgery across the country with a response rate of 74% using mailed follow-up. Although responders and non-responders were similar with regards to their living arrangements, number of comorbidities and baseline health status, responders were more likely to be older, women and of a higher SES. The impact of any response bias appears to be slight. Response bias due to sex could overestimate the improvement in health status by 1% (0.05/4.45) on the GIQLI score and by 9% (0.005/0.060) on the EQ-5D index. In contrast, age bias may underestimate the improvement by 10% (0.5/4.6) on the GIQLI score and by 2% (0.001/0.060) on the EQ-5D.

The mean GIQLI had improved by 3 months from 93.3 to 97.9. This suggests that patients regain their prior level of GI health after major emergency surgery and there is an improvement compared with a month before their emergency admission. GIQLI symptom also improve, by eight when compared with baseline, though GIQLI social decreased by 1.3. Patients’ overall health status measured by the EQ-5D showed a considerable increase (0.58 vs 0.64), although this was not quite statistically significant.

### What this study adds

This study has demonstrated the feasibility of collecting PROMs 3 months after emergency surgery among patients who, during their admission, had supplied retrospective accounts of the pre-event health status. It was shown that with high response rates, any responder bias is slight and will not undermine comparisons of providers. The higher response rates achieved in our study compared with a prior study in England^9^ may reflect the severity of emergency laparotomy as a subset of emergency surgical admissions. In elective surgery, higher response rates are observed with major procedures such as hip replacement than minor procedures such as inguinal hernia repair.^27^ In addition, mailing the questionnaires from a university rather than the hospital created the perception of independent assessment which may have encouraged participation.

The observation that the GIQLI social score worsens despite *the symptom* score improving was unexpected. It may be that the use of retrospective reporting of preoperative symptoms exaggerates their severity though such a bias was not detected in studies of elective surgery.^19 20^ It could be that the GIQLI social score items require a longer recovery trajectory than GIQLI symptom items.

The improvement of generic health status, as seen by the increase in EQ-5D, may reflect that emergency laparotomies are primarily performed in lifesaving situations; the improved health outcomes would imply that these procedures are lifesaving and restorative and also goes further and improves the quality of life of patients. This is not unsurprising, as a proportion of emergency laparotomies will be performed for conditions that may be associated with chronic symptoms prior to acute presentation (such as acute colonic perforation in diverticular disease). As such, recall of symptoms in the month preceding surgery may also encompass the impact of chronic disease.

### Strengths and limitations

This is the first study of using retrospective PROMs to collect patients’ baseline health status and a 3-month follow-up for those admitted for emergency surgical operations in England. It was also conducted in multiple sites (11 hospital trusts) in different regions in England. This confirmed the feasibility of recruiting patients from diverse different geographical populations, as well as assessing PROMs use in different hospital organisational cultures and environments.

There are several limitations. First, some patients did not respond to the GIQLI item on their sexual life as there was no option to report ‘not applicable’. This introduced some response bias. Second, some patients (defined by their cognitive or literacy ability) were not eligible for inclusion in NELA so could not be included in this study.

Third, PROMs have not been widely used in emergency surgery and their psychometric properties (eg, inter-rater reliability) in such patients have not been demonstrated. Among the disease-specific PROMs with adequate measurement properties, the GIQLI is the most commonly used. There is a need for further research in the systematic development of PROMs for use in emergency admissions, including psychometric testing for use in emergency laparotomy.

Fourth, although not the subject of this paper, determining the outcome of emergency laparotomy using PROMs will inevitably depend on the validity of patients’ recall of their pre-event health status. This has been addressed in a previous paper.^21^

Finally, only one follow-up was conducted so there is little insight into the recovery trajectory of emergency laparotomy patients. Additional follow-ups should be considered in future studies.

## Conclusion

This approach assesses from the patient’s perspective the impact of emergency laparotomy treatment. It also offers an insight into the opportunity for assessing other hospital admissions that are emergencies. The generalisability of these findings needs to be investigated with research on other causes of emergency admissions.

Further research is needed in a larger sample of patients to explore longer-term outcomes enabling mapping of recovery trajectories. In addition, by capturing clinical data on patients (eg, P-POSSUM scores), such as by linkage to national clinical audit data, it would be possible to determine any association with indications for surgery, diagnosis and severity. This would be essential to be able to make meaningful comparisons of hospitals’ outcomes and to ensure the PROMs data could support clinical decisions.

Routine collection of PROMs in emergency admissions could be feasible by their inclusion in national clinical audits. Such data would enhance quality improvement by including, alongside clinical outcomes, information on patients’ views of their symptoms, functional status and quality of life. For patients undergoing emergency laparotomy, there is a paucity of information available on the longer-term functional outcomes. Evidence obtained from PROMs can help inform shared decision-making before undertaking potentially high-risk surgery.
