# Supplementary material for: Feasibility of collecting and assessing patient-reported outcomes for emergency admissions: laparotomy for gastrointestinal conditions
Source: BMJ Open Gastroenterol. 2018 Oct 16;5(1):e000238. doi: 10.1136/bmjgast-2018-000238 (PMC6203065; doi:10.1136/bmjgast-2018-000238)

Appendices

Appendix 1: GIQLI Questionnaire [23]

1. Over the past 2 weeks, how often have you had abdominal pains?

all the time,	most of the time,	now and then,	rarely,	never
(0)	(1)	(2)	(3)	(4)

2. Over the past 2 weeks, how often have you been bothered by a feeling of fullness in the upper abdomen?

all the time,	most of the time,	now and then,	rarely,	never
(0)	(1)	(2)	(3)	(4)

3. Over the past 2 weeks, how often have you felt bothered by bloating or the sensation of having too much gas in the abdomen?

all the time,	most of the time,	now and then,	rarely,	never
(0)	(1)	(2)	(3)	(4)

4. Over the past 2 weeks, how often have you felt bothered by passing wind?

all the time,	most of the time,	now and then,	rarely,	never
(0)	(1)	(2)	(3)	(4)

5. Over the past 2 weeks, how often have you felt bothered by burping or belching?

all the time,	most of the time,	now and then,	rarely,	never
(0)	(1)	(2)	(3)	(4)

6. Over the past 2 weeks, how often have you noticed unusual stomach or bowel noises?

all the time,	most of the time,	now and then,	rarely,	never
(0)	(1)	(2)	(3)	(4)

7. Over the past 2 weeks, how often have you been bothered by frequent bowel movements?

all the time,	most of the time,	now and then,	rarely,	never
(0)	(1)	(2)	(3)	(4)

8. Over the past 2 weeks, how often have you really enjoyed eating?

all the time,	most of the time,	now and then,	rarely,	never
(4)	(3)	(2)	(1)	(0)

9. How often have you had to refrain from eating the food you love due to your illness?

all the time,	most of the time,	now and then,	rarely,	never
(0)	(1)	(2)	(3)	(4)

10. Over the past 2 weeks, how did you manage to cope with everyday stress?

very badly,	badly,	moderately,	well,	very well
(0)	(1)	(2)	(3)	(4)

11. Over the past 2 weeks, how often have you felt sad about the fact that you are sick?

all the time,	most of the time,	now and then,	rarely,	never
(0)	(1)	(2)	(3)	(4)

12. Over the past 2 weeks, how often have you been nervous or anxious because of your illness?

all the time,	most of the time,	now and then,	rarely,	never
(0)	(1)	(2)	(3)	(4)

13. Over the past 2 weeks, how often have you been satisfied with your life in general?

all the time,	most of the time,	now and then,	rarely,	never
(4)	(3)	(2)	(1)	(0)

14. Over the past 2 weeks, how often have you felt frustrated about your illness?

all the time,	most of the time,	now and then,	rarely,	never
(0)	(1)	(2)	(3)	(4)

15. Over the past 2 weeks, how often have you felt tired or weary?

all the time,	most of the time,	now and then,	rarely,	never
(0)	(1)	(2)	(3)	(4)

16. Over the past 2 weeks, how often have you felt unwell?

all the time,	most of the time,	now and then,	rarely,	never
(0)	(1)	(2)	(3)	(4)

17. Over the past week (past 7 days), how many nights did you wake up at least once during the night?

every night,	5 to 6 nights,	3 to 4 nights,	1 to 2 nights,	never
(0)	(1)	(2)	(3)	(4)

18. To what extent has your illness led to disturbing changes in your appearance?

very much,	much,	somewhat,	a little,	not at all
(0)	(1)	(2)	(3)	(4)

19. To what extent has your general physical strength deteriorated due to your illness?

very much,	much,	moderately,	a little,	not at all
(0)	(1)	(2)	(3)	(4)

20. To what extent have you lost your stamina due to your illness?

very much,	much,	moderately,	a little,	not at all
(0)	(1)	(2)	(3)	(4)

21. To what extent have you lost your fitness due to your illness?

very much,	much,	moderately,	a little,	not at all
(0)	(1)	(2)	(3)	(4)

22. Over the past two weeks, have you been able to continue your normal daily activities (such as work, school, and household tasks)?

all the time,	most of the time,	now and then,	rarely,	never
(4)	(3)	(2)	(1)	(0)

23. Over the past 2 weeks, have you been able to continue your normal recreational activities (such as sports and hobbies)?

all the time,	most of the time,	now and then,	rarely,	never
(4)	(3)	(2)	(1)	(0)

24. Over the past 2 weeks, have you felt very restricted by the medical treatment?

all the time,	most of the time,	now and then,	rarely,	never
(0)	(1)	(2)	(3)	(4)

25. To what extent have your relationships with people close to you changed due to your illness?

very much,	much,	moderately,	a little,	not at all
(0)	(1)	(2)	(3)	(4)

26. To what extent has your sex life been impaired by your illness?

very much,	much,	moderately,	a little,	not at all
(0)	(1)	(2)	(3)	(4)

27. Over the past 2 weeks, have you been bothered by regurgitation of fluid or food?

all the time,	most of the time,	now and then,	rarely,	never
(0)	(1)	(2)	(3)	(4)

28. Over the past 2 weeks, how often have you felt bothered by your slow eating?

all the time,	most of the time,	now and then,	rarely,	never
(0)	(1)	(2)	(3)	(4)

29. Over the past 2 weeks, how often have you felt bothered by difficulty swallowing your food?

all the time,	most of the time,	now and then,	rarely,	never
(0)	(1)	(2)	(3)	(4)

30. Over the past two weeks, how often have you been bothered by urgent bowel movements?

all the time,	most of the time,	now and then,	rarely,	never
(0)	(1)	(2)	(3)	(4)

31. Over the past 2 weeks, how often have you been bothered by diarrhoea?

all the time,	most of the time,	now and then,	rarely,	never
(0)	(1)	(2)	(3)	(4)

32. Over the past 2 weeks, how often have you been bothered by constipation?

all the time,	most of the time,	now and then,	rarely,	never
(0)	(1)	(2)	(3)	(4)

33. Over the past 2 weeks, how often have you been bothered by nausea?

all the time,	most of the time,	now and then,	rarely,	never
(0)	(1)	(2)	(3)	(4)

34. Over the past 2 weeks, how often have you been alarmed by blood in your stool?

all the time,	most of the time,	now and then,	rarely,	never
(0)	(1)	(2)	(3)	(4)

35. Over the past 2 weeks, how often have you been bothered by heartburn?

all the time,	most of the time,	now and then,	rarely,	never
(0)	(1)	(2)	(3)	(4)

36. Over the past 2 weeks, how often have you been bothered by involuntary bowel movements?

all the time,	most of the time,	now and then,	rarely,	never
(0)	(1)	(2)	(3)	(4)

GIQLI © Ernst Eypasch, 1995. All Rights Reserved

Appendix 2: Study Flow Diagram

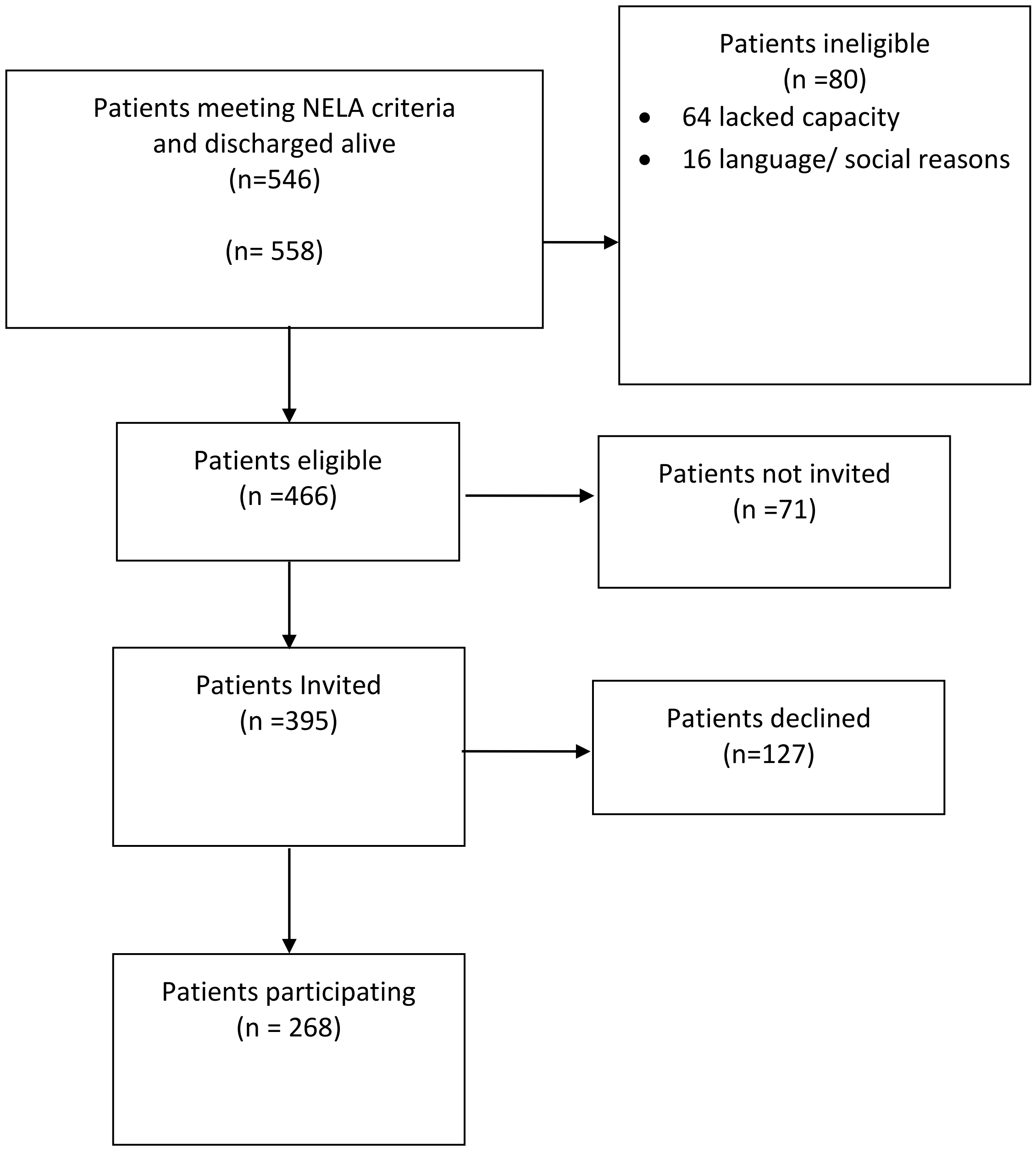

Supplement: Supplementary data [file bmjgast-2018-000238supp001.pdf]
